# A Treatise on Diseases of the Nose and Throat

**Published:** 1890-02

**Authors:** 


A **Treatise on Diseases of the Nose and Throat**, in two volumes, by FRANCKE HUNTINGTON BOSWORTH, A. M., M. D., Professor of Diseases of the Throat, Bellevue Hospital Medical College, New York; Fellow of the American Laryngological Association, of the American Climatological Association, etc., etc. Volume I., Diseases of the Nose and Naso-Pharynx, with four colored plates and 182 wood cuts. Cloth; octavo, pp. xvii., 670. New York: William Wood & Co. 1889.

This is an important work, and demands careful examination. It is a new work, for, although Dr. Bosworth published in 1881 a volume on the diseases of the throat and nose, the present one has been written almost without reference to it, and contains but two chapters in any way suggestive of the earlier publication. The author is an enthusiastic student and expresses his opinions with force and clearness. He has done much to advance the knowledge of the diseased conditions, as seen in the upper air passages, and even where his opinions have not been entirely accepted, the very directness with which they have been stated has stimulated inquiry, and has often led to a reëxamination of the subject. When, therefore, it was known that Dr. Bosworth was preparing a new work on the diseases of these organs, it was expected that it would be in the largest sense original. In this we have not been disappointed. It is one merit of this volume that it gives the experience of an observer who has had large opportunities, and who has used them as only a student can. It is not too much to say that laryngological literature is the richer for the book, or that the workers in the same field will find in it much that will be of great assistance to them.

The opening chapters relate to Methods of Examination, Methods of Treatment, Mucous Membranes, Taking Cold, the Anatomy and Physiology of the Nose, and Some General Considerations concerning Catarrhal Diseases. These chapters illustrate a particular merit of the author's writing—that of simplicity. The study of disease and its treatment is a complicated one at its best, and it does not seem necessary to make it more so by involved processes of reasoning. Some idea of this kind the author probably had, for we are continually meeting sentences condemning elaborate methods of investigation. On page 11, for instance, he says: "In other words, a simple head-band on the forehead, and a good strong source of light, afford us in every respect as good a method of practising laryngoscopy as the most elaborate apparatus." And this statement is repeated on page 13. In many places complicated methods of treatment are condemned, and the simpler ones advocated. The ordinary hand-ball atomizer, with the single bulb, answers as well as the air pump and receiver. The latter is a convenience, but not necessary. The chapter on taking cold contains a great deal of excellent advice. It ought to be published in pamphlet form, so that the body of the profession not likely to consult a special work could become familiar with it. The study of the various diseases begins with a chapter on acute rhinitis. This chapter should be read in connection with the one just mentioned, that on taking cold, and we feel sure if the suggestions contained in these two chapters were followed—not simply read and forgotten—there would be fewer colds, and consequently less trouble with the upper air passages. The subject of Hypertrophic Rhinitis receives full and careful consideration. Dr. Bosworth protests against the habit often seen in European writers, of speaking of catarrh as an American disease. This assertion is, to put it mildly, a rather loose one. If the cases were carefully examined that presented themselves for treatment for throat disease, many of them would be found to be suffering from nasal disease as well, and, indeed, the latter is often the direct cause of the former. The evolution of a catarrh results from a complex series of causes and is seen almost everywhere. It cannot be put aside by simply calling it an American disease. We may know more about it in this country than they do in some others, but that is because we have studied it more, not because as a Nation it is more prevalent here.

The subject of the Purulent Rhinitis of Children is a very important one, especially as it is often the first step in the development of the atrophic rhinitis. Our observation tends to confirm the author's opinion on this point. The latter disease is so very obstinate that any suggestions in its prevention is of great value. The appearance of a purulent rhinitis in a child, therefore, demands attention at once, for by proper treatment the atrophic condition in later life can be prevented. In this connection Dr. Bosworth records an interesting clinical observation, to the effect that in early life the mucous structures seem prone to pathological processes, while in adult life it is the connective tissues that exhibit this tendency. This will explain some of the differences in the diseases seen in these different periods.

We have a short chapter on that rare, but interesting, disease called Croupous Rhinitis, in which a false membrane forms on the mucous surface, and which is very obstinate, not yielding readily to treatment. The author believes more in the constitutional than in the local treatment, though both are necessary.

The question of Nasal Reflexes receives extended treatment. In this notice we can but call attention to the importance of this subject, and to the excellent manner in which it is handled by the author. The general physician who comes in contact with all sorts of cases daily, will find here much valuable information that will help him in obscure cases. It would be well if he were familiar with it. The specialists will consult it often, and will not be disappointed in the thoroughness with which the various reflexes are analyzed and discussed. The views of Dr. Bosworth upon Hay Fever or Vaso-motor Rhinitis and Asthma or Vaso-motor Bronchitis, are very well known, and have occasioned more or less controversy. They are very fully discussed here.

Hay fever depends upon three factors, namely:

I. The presence of pollen in the atmosphere.

II. A neurotic habit.

III. A local morbid condition of the nasal mucous membrane.

Asthma also depends upon three factors:

I. A general neurotic condition.

II. A diseased condition of the nasal mucous membrane (not the bronchial.)

III. Some obscure condition of the atmosphere exciting the paroxysms.

It will be observed that one of the factors in each disease is a morbid process in the nasal passages. Whether we carry this proposition as far as the author or not, there is, no doubt, a large amount of truth in his position which we must recognize and act upon. It is, therefore, necessary to have each of these cases examined as to the condition of the nose, so as to be sure not to overlook this important complication. When found to exist, it, of course, must be removed before we can look for a successful termination of the case. It is a new departure to find a chapter on asthma in a work upon the nose, and yet we think the author was justified in so including it. We believe, also, that much good will result from the stimulus he has given to the study of this disease, which cannot help but be of material benefit to patients.

We have next short chapters on Nasal Hydrorrhea and Anosmia, and then we come to a chapter of especial interest on Deformities of the Nasal Septum. Here, again, the athor has done a great deal of original work. We must accept to-day as the result of his teaching that it is absolutely necessary to correct these deformities in order to relieve the cases. He believes that they are probably the most frequent of all the exciting causes of catarrhal inflammation of the nasal mucous membrane—a position that is not easily disputed. In the removal of these deformities the author prefers the saw, an opinion in which we concur, though sometimes it must be abandoned for other means. The illustrations in this chapter are very good, and serve to clearly explain the text.

The following subjects are then considered in their order: Epistaxis, Foreign Bodies in the Nasal Passages, Rhinoliths, Parasites in the Nasal Cavities, Syphilis of the Nasal Passages, and also its Congenital Manifestation, Tuberculosis and Lupus of the Nasal Passages, and Rhinoscleroma.

In a series of ten chapters are considered next the various neoplasms as seen within the nose, beginning with the ordinary nasal polyp and ending with carcinoma. While some of these forms are very rare, others are comparatively common, and we find that they are presented here in a manner that leaves nothing to be desired. The special student will not find wanting anything that would serve to make him familiar with any question likely to arise concerning these growths, especially with their diagnosis and treatment.

The section closes with a chapter on Diseases of the Accessory Sinuses of the Nose. Separate divisions consider the diseases of the Autrum, the Ethmoidal Sinuses, the Sphenoidal Sinuses, the Frontal Sinuses, and the Differential Diagnosis between Diseases of the Accessory Cavities. A knowledge of these subjects is absolutely essential to the specialist, and we find them presented here in a concise manner and with a clear appreciation of the various conditions. It is a valuable chapter for reference and will probably be consulted as much as any part of the work. The next section considers the Diseases of the Naso-Pharynx. While this region is generally involved in any morbid process beginning in the nose, there are certain conditions seen here alone. This is especially so with some of the neoplastic formations and with Hypertrophy of the Pharyngeal Tonsil, or as it is sometimes called, Adenoid growths in the pharyngeal vault. The chapter dealing with the last mentioned disease deserves special notice. It gives a full account of our knowledge concerning it, together with the various methods of treatment. In the operative treatment Dr. Bosworth prefers the cold wire snare where it can be used—though, of course, he recognizes that there are many cases where some other method must be employed.

The chapter on Fibroma of the Naso-Pharynx is another that will be frequently consulted. This often very serious disease must be recognized early and the treatment must be radical. The methods the author considers best are the cold wire snare and electrolysis. Detailed descriptions of these operations are given.

The third and last section of the work treats of the External Surgery of the Nose, and describes those operations by which larger access to the nasal passages is obtained for the removal of tumors. These operations are of three classes, and include:

I. Incisions through the hard or soft palate, or both.

II. Incisions through the external integument alone.

III. Incisions through the external integument together with section of bone.

Under these various groups are described no less than thirty-three operations, each bearing the name of the surgeon first to propose it. The section is beautifully illustrated with four colored plates and many wood cuts, which served to make the text plain. This part is as elaborate as any we have yet passed in review, and materially contributes to the value of the book. While some specialists would no doubt prefer that these operations should be performed by a general surgeon, they should all know wherein they differ from one another and their relative value.

The work is completed by a Biographical and a General Index.

It will be seen that this is an exhaustive treatise, and one that the specialist, at least, cannot afford to be without. The publisher's work is well done. The type is large and clear, and the illustrations finely executed. Take it all in all, this work stands easily among the first ever published in this or any country, upon the subjects of which it treats. We await with interest the second volume, which we understand is to follow shortly.

